# Metformin for endometrial hyperplasia: a Cochrane protocol

**DOI:** 10.1136/bmjopen-2016-013385

**Published:** 2016-08-15

**Authors:** Naomi S Clement, Thomas R W Oliver, Hunain Shiwani, Juliane R F Saner, Caroline A Mulvaney, William Atiomo

**Affiliations:** 1 Faculty of Health Sciences and Medicine, Queen's Medical Centre, University of Nottingham, Nottingham, UK; 2 Division of Surgery, Watford General Hospital, Watford, UK; 3 Faculty of Health Sciences and Medicine, Leeds Institute of Medical Education, Leeds, UK; 4 Faculty of Health Sciences and Medicine, Care of the Elderly, Epsom and St Helier University Hospitals Trust, Carshalton, UK; 5 Research Design Service, School of Medicine, University of Nottingham, Nottingham, UK

**Keywords:** GYNAECOLOGY, CLINICAL PHARMACOLOGY

## Abstract

**Introduction:**

Endometrial hyperplasia is a precancerous lesion of the endometrium, commonly presenting with uterine bleeding. If managed expectantly, it frequently progresses to endometrial carcinoma, rates of which are increasing dramatically worldwide. However, the established treatment for endometrial hyperplasia (progestogens) involves multiple side effects and leaves the risk of recurrence. Metformin is the most commonly used oral hypoglycaemic agent in type 2 diabetes mellitus. It has also been linked to the reversal of endometrial hyperplasia and may therefore contribute to decreasing the prevalence of endometrial carcinoma without the fertility and side effect consequences of current therapies. However, the efficacy and safety of metformin being used for this therapeutic target is unclear and, therefore, this systematic review will aim to determine this.

**Methods and analysis:**

We will search the following trials and databases with no language restrictions: Cochrane Gynaecology and Fertility Specialised Register; Cochrane Central Register of Controlled Trials (CENTRAL); MEDLINE; EMBASE; EBSCO Cumulative Index to Nursing and Allied Health Literature; PubMed; Google Scholar; ClinicalTrials.gov; the WHO International Trials Registry Platform portal; OpenGrey and the Latin American and Caribbean Health Sciences Literature (LILACS). We will include randomised controlled trials (RCTs) of use of metformin compared with a placebo or no treatment, conventional medical treatment (eg, progestogens) or any other active intervention. Two review authors will independently assess the trial eligibility, risk of bias and extract appropriate data points. Trial authors will be contacted for additional data. The primary review outcome is the regression of endometrial hyperplasia histology towards normal histology. Secondary outcomes include hysterectomy rate; abnormal uterine bleeding; quality of life scores and adverse reactions to treatments.

**Ethics and dissemination:**

Dissemination of the completed review will be through the Cochrane Library as well as through presenting the results at appropriate conferences.

**Strengths and limitations of this study**

- This is the first systematic review to scrutinise the combined power of existing studies to examine the effect of metformin on endometrial hyperplasia.
- The use of the systematic approach in evaluating randomised controlled trials through the Cochrane system will provide a definite, comprehensive answer to this question (Cochrane ID CD012214).
- Limitations will be due to the biases present in selected studies. This will be overcome by analysing the risk of bias in all selected studies.

## Introduction

### Description of the condition

Endometrial hyperplasia is a precancerous endometrial lesion that commonly presents with abnormal uterine bleeding. It is thought to be due to unopposed, prolonged exposure of the endometrium to oestrogen; if managed expectantly, it can progress to endometrial carcinoma, although the condition may resolve spontaneously. It is diagnosed histologically and can be subsequently categorised into four subtypes: simple, simple with atypia, complex and complex with atypia.^1^ Risk of progression to endometrial carcinoma is dependent on the type of endometrial hyperplasia, and progression rates vary widely across the literature. This discrepancy is likely due, in part, to the fact that many cases of endometrial hyperplasia, especially when atypia is present, are managed pre-emptively with a hysterectomy. However, atypia is thought to be a strong risk factor for progression to adenocarcinoma.^1^ Progression rates have been reported as <5% for non-atypical hyperplasia but 28% for atypical hyperplasia cumulatively over 20 years. This difference in progression risk has also been seen at interval-specific time points of 4, 9 and 20 years postdiagnosis.^2^ Risk factors for endometrial hyperplasia are, predictably, very similar to those for endometrial carcinoma and include obesity, diabetes mellitus, nulliparity, tamoxifen use, oestrogen therapy and polycystic ovarian syndrome (PCOS).^3^

PCOS is a metabolically driven gynaecological disorder thought to affect 10% of women of childbearing age.^4^ A diagnosis of PCOS must fulfil the widely accepted Rotterdam criteria of two or more of the following in the absence of another cause of chronic anovulation: hyperandrogenism (clinical or biochemical), chronic oligo/anovulation and polycystic ovaries apparent on ultrasound.^5^ The prevalence of endometrial hyperplasia in women with PCOS varies greatly in the literature—between 1% and 48.8%,^6–9^ but risk of endometrial carcinoma is well founded, as women with PCOS possess a threefold increased risk of developing endometrial carcinoma when compared with the non-PCOS population.^10^

The aim of endometrial hyperplasia treatment, whether or not PCOS is a comorbidity, is to control abnormal vaginal bleeding while minimising risk of progression to endometrial carcinoma. Historically, endometrial hyperplasia without atypia has been medically treated with oral progestogens (alone or in combination with oestrogen in PCOS) or intrauterine progestogens, inhibiting oestrogen-driven cell growth and inducing withdrawal bleeds.^11^ This treatment provides the benefit of preserving fertility but is associated with side effects—in the short term, headaches, mood changes, acne or breast tenderness, and over the longer term, risk of a thromboembolic event or breast cancer. These longer term side effects can be mitigated by educating women on the symptoms of thromboembolic events and by ensuring that they attend regular breast cancer screening programmes. This approach has the effect of potentially hindering compliance, consequently producing a relatively high relapse rate. In one study, 30.3% and 13.7% of women treated with oral progestogens and intrauterine levonorgestrel, respectively, had relapse of their endometrial hyperplasia.^12^ In women with atypia and in those who are resistant to progestogens, surgical hysterectomy is the treatment of choice.

### Description of the intervention

Metformin, a biguanide that acts as an insulin sensitiser, is the most commonly used oral hypoglycaemic agent in type 2 diabetes mellitus. It acts to inhibit hepatic gluconeogenesis, decreasing liver glucose production and thereby decreasing levels of circulating glucose and insulin.

Metformin is also prescribed for women with PCOS to induce weight loss and improve menstrual regularity, both as monotherapy and in combination with a progestogen. It is frequently used to treat ovulation dysfunction in women with PCOS when they have shown resistance to treatment with clomiphene. Despite widespread use of metformin in women with PCOS, no definitive improvement in clinical or biochemical features has been shown on systematic review when metformin is compared with the contraceptive pill.^13^ It has an established side effect profile, including nausea and vomiting, diarrhoea, abdominal pain and changes in taste, as well as rarer or less publicised effects including lactic acidosis or decreased B_12_ absorption, possibly leading to anaemia and potentially irreversible neuronal damage if left unmonitored and uncorrected for prolonged periods.^14^

### How the intervention might work

Hyperinsulinaemia secondary to insulin resistance is thought to exhibit a mitogenic effect, inducing cell division via mitosis—a risk factor for hyperplasia—and, ultimately, carcinoma development. This effect is likely due to its activity at the insulin-like growth factor-1 receptor, promoting proliferation and angiogenesis, which can be demonstrated by the positive correlation between diabetes and breast and gynaecological cancers.^15^ Insulin-mediating effects of metformin, then, show evidence of reducing incidence and improving survival among these malignancies, although the evidence is mixed.^16^ ^17^ The link between insulin resistance and cell proliferation offers an intriguing potential therapeutic target to reverse hyperplasia and prevent endometrial carcinoma. Some early trials have corroborated this link, showing efficacy of metformin in inducing endometrial atrophy in benign endometrial proliferative disorders; one reported atrophy and, therefore, reversal of endometrial hyperplasia in 96% of women treated with metformin.^18^

Other proposed mechanisms of the anticancer properties of metformin include its direct effects on cell signalling pathways, including inhibition of the mammalian target of rapamycin (mTOR) and inhibition of mitogen-activated protein kinase (MAPK) and Akt activity. These pathways are involved in cell proliferation and therefore play a key role in hyperplasia and cancerous lesions in any tissue. As metformin inhibits these pathways, cell proliferation will be hindered, reducing the chance of development of cancerous lesions.^19^ ^20^

### Why it is important to do this review

Current medical therapy for endometrial hyperplasia involves multiple side effects and leaves the risk of recurrence. Therefore, a systematic review of a novel, alternative therapy is needed to collate the evidence to date and guide future human trials. Risk of progression from endometrial hyperplasia to carcinoma is significant; up to 40% of women suffering from endometrial hyperplasia with atypia go on to develop carcinoma, the most common fatal gynaecological malignancy.^21^ This rate is expected to increase by up to 100% globally over the next 20 years.^22^ The biguanide insulin sensitiser metformin has been linked to reversal of endometrial hyperplasia^18^ and, if it can be used in this way, may contribute to decreasing the prevalence of endometrial carcinoma without leading to the fertility consequences of current therapies. Metformin is also used as an alternative therapy in women with PCOS, among whom risk of endometrial hyperplasia is increased. However, the mode of action, efficacy and safety of metformin remain unclear. This review may help to clarify its role in the treatment of women with this disease.

## Objective

To determine the efficacy and safety of metformin in treating women with endometrial hyperplasia.

## Methods and analysis

### Criteria for considering studies for this review

#### Types of studies

The review will consider only randomised controlled trials (RCTs), published and unpublished, as eligible for inclusion. We will include cross-over trials, but we will use in the analysis only data from the first phase, as cross-over is not a valid study design for the purposes of this review.

#### Types of participants

We will select women who have histologically confirmed endometrial hyperplasia of any type as the study population of the review.

#### Types of interventions

We will include trials of metformin compared with placebo or no treatment, conventional medical treatment (typically progestogens, eg, oral or intrauterine) or any other active intervention. We will include trials that provide cointerventions (eg, metformin plus progesterone vs progesterone) but will analyse these studies separately.

#### Types of outcome measures

Primary outcomes:

- Regression of endometrial hyperplasia histology (with or without atypia) towards normal histology

Secondary outcomes:

- Recurrence of endometrial hyperplasia
- Progression of endometrial hyperplasia to endometrial cancer
- Hysterectomy rate
- Abnormal uterine bleeding
- Health-related quality of life, as reported in the included study
- Adverse effects during treatment, as reported in the included study

We will report outcomes measured after short-term treatment (up to 6 months post-treatment), medium-term treatment (6–12 months post-treatment) and long-term treatment (>12 months post-treatment).

### Search methods for identification of studies

We will search for all published and unpublished RCTs of metformin for endometrial hyperplasia without language restriction. Review authors will liaise with the Cochrane Gynaecology and Fertility Group Trials search coordinator when conducting the search.

#### Electronic searches

In accordance with guidance from the Cochrane Gynaecology and Fertility Group, we will create search strategies for the following electronic databases to identify all relevant RCTs.

- Cochrane Gynaecology and Fertility Specialised Register (inception to present) (see online supplementary appendix 1)
- Cochrane Central Register of Controlled Trials (Ovid CENTRAL) (inception to present) (see online supplementary appendix 2)
- Ovid MEDLINE (inception to present) (see online supplementary appendix 3)
- Ovid EMBASE (inception to present) (see online supplementary appendix 4)
- EBSCO Cumulative Index to Nursing and Allied Health Literature (CINAHL) (inception to present) (see online supplementary appendix 5)
- PubMed (inception to present) (see online supplementary appendix 6)
- Google Scholar (inception to present) (see online supplementary appendix 7)

We will perform a further search to identify ongoing or unpublished trials related to the review.

- ClinicalTrials.gov (inception to present) (see online supplementary appendix 8)
- WHO International Trials Registry Platform search portal (inception to present) (see online supplementary appendix 9)
- OpenGrey (inception to present) (see online supplementary appendix 10)
- Latin American and Caribbean Health Sciences Literature (LILACS) (inception to present) (see online supplementary appendix 11)

We will present a list of search strings in the appendices and will email the contact persons of all unpublished trials identified to assess these studies for potential inclusion.

10.1136/bmjopen-2016-013385.supp1 Supplementary appendices

## 

### 

#### Searching other resources

To identify additional trials, we will handsearch the bibliographies of all included studies, as well as any reviews on the topic. We will also handsearch the European Society of Human Reproduction and Embryology (ESHRE) 2015 and the American Society for Reproductive Medicine (ASRM) 2015 conference abstracts for relevant presentations. Previous abstracts from these conferences are already incorporated into the Cochrane Gynaecology and Fertility Specialised Register.

### Data collection and analysis

#### Selection of studies

We will add to a reference manager (Covidence) the titles and abstracts of all studies retrieved by electronic searches and will remove duplicates. Two review authors (NC, TRWO, JRFS, HS) will independently review each entry and will assess titles and abstracts for potential inclusion in the review. We will seek full-text reports for potentially relevant studies. Again, two review authors (NC, TRWO, JRFS, HS) will independently assess each full-text report against the inclusion criteria and will document a justification for rejection of each study. Review authors will resolve disagreements between them regarding trial suitability by discussion or by consultation with a third review author. We will screen studies for duplicate publication by comparing study author names, locations, dates and durations. When uncertainty about study methods or the possibility of duplicate studies arises, we will contact the authors of relevant papers.

We will construct a flow chart to illustrate selection of studies for inclusion in this review according to Preferred Reporting Items for Systematic Reviews and Meta-Analyses (PRISMA) guidelines.^23^

#### Data extraction and management

Two review authors will independently extract data using a data extraction form that is based on the ‘Checklist of items to consider in data collection or data extraction’ provided in the *Cochrane Handbook for Systematic Reviews of Interventions.*^24^ During study selection, if we find a study that has been published multiple times, we will extract and collate the data into a single file. We will treat such studies as a single unit of interest for the review and will attribute multiple references to the single file.

When necessary, we will liaise with study authors to obtain additional data on their methods and/or results.

#### Assessment of risk of bias in included studies

Two review authors (from NC, TRWO, HS or JRFS) will independently assess each included study for risk of bias by using the Cochrane ‘Risk of bias’ assessment tool, as described in the *Cochrane Handbook for Systematic Reviews of Interventions.*^24^ We will categorise bias in the following manner.

- Selection bias (random sequence generation and allocation concealment)
- Performance bias (blinding of participants and personnel)
- Detection bias (blinding of outcome assessments)
- Attrition bias (incomplete outcome data)
- Reporting bias (selective reporting)
- Other bias (other sources of bias)

We will classify risk of bias as ‘low’, ‘high’ or ‘unclear’ for all domains mentioned above by using the ‘Criteria for judging risk of bias’ in the ‘Risk of bias’ assessment tool.^24^ We will resolve disagreements by discussion and when necessary by consultation with a third review author. We will fully justify judgements made and will include this information in the ‘Risk of bias’ table. We will account for findings of this assessment when we interpret findings of the review, as when performing the sensitivity analysis. We will report the level of risk chosen and evidence used to make that judgement, for example, quotes from the text, in a ‘Characteristics of included studies’ table. To minimise bias in selective reporting of trial outcomes, when possible, we will compare published protocols versus methods described in the final study.

#### Measures of treatment effect

For dichotomous data (eg, regression of endometrial hyperplasia, progression to endometrial carcinoma), we will calculate the Mantel-Haenszel OR from the numbers of events in control and intervention groups.

For continuous data, we will use means, SDs and mean differences (MDs). We will treat ordinal data, such as side effect severity scoring systems or health-related quality of life questionnaires, as continuous data for purposes of analysis. When different scales are used to report similar outcomes (eg, change in endometrial thickness), we will calculate the standardised MD (SMD). We will express the SMD effect as small (0.2 to <0.5), medium (0.5 to <0.8) or large (≥0.8).

We will provide 95% CIs for all outcomes.

When ORs and MDs cannot be derived from the data, we will perform an alternative statistical analysis of included studies when possible. We will report the values produced by analysis in full, along with the direction and magnitude of effect of the intervention, and whether or not findings are statistically significant.

#### Unit of analysis issues

We will perform the primary analysis per woman. When a valid analysis is not possible (eg, ‘per cycle’ data), we will briefly summarise the data but will not include them in the meta-analysis. We will include in the analysis only first-phase data from cross-over trials.

#### Dealing with missing data

We will analyse only available data. When contacting study authors for missing information, we will send a first reminder email 14 days and a second reminder email 21 days after the initial email. Should we determine that data are missing, we will address the potential impact of this fact in the Discussion section of the review.

#### Assessment for heterogeneity

We will consider whether clinical and methodological characteristics of included studies are sufficiently similar for meta-analysis to provide a clinically meaningful summary. We will assess statistical heterogeneity by using the measure of I^2^. We will consider I^2^>50% to indicate substantial heterogeneity.^24^ ^25^

#### Assessment of reporting bias

Reporting bias is a potential issue for all reviews. We will aim to identify and minimise reporting bias in our analysis by creating a comprehensive search strategy and using a multitude of electronic databases, including those that record unpublished work and work prepared in languages other than English. This should ensure that we maximise the yield of eligible studies included in the review and identify cases of data duplication.

If we include 10 or more studies in a single analysis, we will use a funnel plot to explore the possibility of small-study effects (ie, the tendency for estimates of the intervention effect to be more beneficial in smaller studies).

#### Data synthesis

If identified studies are sufficiently similar, we will combine the data using a fixed-effects model for the following comparisons.

- Metformin versus placebo or no treatment
- Metformin versus progestogens
- Metformin versus other active intervention
- Metformin plus cointervention versus cointervention alone

We will stratify analyses by dose of metformin (high, moderate, low).

We will graphically display results of these meta-analyses, with increasing odds (regardless of whether the outcome is beneficial) demonstrated by a marker right of the centre line and decreasing odds by a marker left of the centre line.

#### Subgroup analysis and investigation of heterogeneity

When data are available, we will conduct subgroup analyses to determine separate evidence within the following subgroups.

- Women with PCOS
- Women with atypical endometrial hyperplasia

Should pooled data demonstrate substantial heterogeneity (>50%), we will consider additional subgroup analyses (eg, by dose or route of metformin) and/or sensitivity analyses. We will acknowledge the degree of heterogeneity when interpreting the meta-analysis.

#### Sensitivity analysis

We will conduct a sensitivity analysis for the primary outcome to determine whether the conclusions are robust to our choice of methods with regards to study eligibility and analysis. Through this sensitivity analysis, we will explore whether the review conclusions would have been different if:

- all studies with high risk of bias in one or more domains were excluded from the analysis;
- a random effects model had been implemented; or
- the effect estimate had been expressed as risk ratio (RR) rather than OR.

#### Overall quality of the body of evidence: summary of findings table

We will prepare a ‘Summary of findings’ table by using GRADEpro Guideline Development Tool software. In this table, we will present a concise overview of the quality of available evidence pertaining to the review outcomes (regression of endometrial hyperplasia towards normal histology, recurrence of endometrial hyperplasia, progression of endometrial hyperplasia to endometrial cancer, hysterectomy rate, abnormal uterine bleeding, health-related quality of life, as reported in included studies and adverse effects during treatment as reported in included studies). In accordance with the Grading of Recommendations, Assessment, Development and Evaluations (GRADE) Working Group criteria (study limitations, consistency of effect, imprecision, indirectness and publication bias), we will rate the quality of the evidence as ‘high’, ‘moderate’, ‘low’ or ‘very low’. We will document the justification for each grade awarded and will incorporate the overall grade into the final conclusion drawn from the results.

## Ethics and dissemination

Owing to this being a systemic review, no ethical permissions are required; however, results of the completed protocol will be completed through the Cochrane Library as well as presented at any appropriate conferences by the authors.
