# Supplementary material for: Metformin for endometrial hyperplasia: a Cochrane protocol
Source: BMJ Open. 2016 Aug 15;6(8):e013385. doi: 10.1136/bmjopen-2016-013385 (PMC5013431; doi:10.1136/bmjopen-2016-013385)
Supplement: Supplementary appendices [file bmjopen-2016-013385supp_appendices.pdf]

Appendix 1

Gynaecology and Fertility Specialised Register search stratergy

Keywords CONTAINS "endometrial hyperplasia" or "endometrial proliferation" or "endometrial thickness" or "proliferation" or "hyperplasia" or Title CONTAINS "endometrial hyperplasia" or "endometrial proliferation" or "endometrial thickness" or "proliferation" or "hyperplasia"

AND

Keywords CONTAINS "metformin" or "glucophage" or Title CONTAINS "metformin" or "glucophage"

Appendix 2

CENTRAL search strategy

1. exp Endometrial Hyperplasia/

2. (endometri$ adj5 hyperplas$).tw.

3. (endometri$ adj3 proliferat$).tw.

4. or/1-3

5. exp Metformin/

6. metformin.tw.

7. glucophage.tw.

8. (dimethylbiguanidine or dimethylguanylguanidine).tw.

9. (dimethylbiguanidium or glucovance).tw.

10. or/5-9

11. 4 and 10

Appendix 3

MEDLINE search strategy

1. exp Endometrial Hyperplasia/

2. (endometri$ adj5 hyperplas$).tw.

3. (endometri$ adj3 proliferat$).tw.

4. or/1-3

5. exp Metformin/

6. metformin.tw.

7. glucophage.tw.

8. (dimethylbiguanidine or dimethylguanylguanidine).tw.

9. (dimethylbiguanidium or glucovance).tw.

10. or/5-9

11. 4 and 10

12. randomized controlled trial.pt.

13. controlled clinical trial.pt.

14. randomized.ab.

15. randomised.ab.

16. placebo.tw.

17. clinical trials as topic.sh.

18. randomly.ab.

19. trial.ti.

20. (crossover or cross-over or cross over).tw.

21. or/12-20

22. exp animals/ not humans.sh.

23. 21 not 22

24. 11 and 23

Appendix 4

EMBASE search strategy

1. exp endometrium hyperplasia/

2. (endometri$ adj5 hyperplas$).tw.

3. (endometri$ adj3 proliferat$).tw.

4. or/1-3

5. exp metformin/

6. metformin.tw.

7. glucophage.tw.

8. (dimethylbiguanidine or dimethylguanylguanidine).tw.

9. (dimethylbiguanidium or glucovance).tw.

10. or/5-9

11. 4 and 10

12. Clinical Trial/

13. Randomized Controlled Trial/

14. exp randomization/

15. Single Blind Procedure/

16. Double Blind Procedure/

17. Crossover Procedure/

18. Placebo/

19. Randomi?ed controlled trial$.tw.

20. Rct.tw.

21. random allocation.tw.

22. randomly.tw.

23. randomly allocated.tw.

24. allocated randomly.tw.

25. (allocated adj2 random).tw.

26. Single blind$.tw.

27. Double blind$.tw.

28. ((treble or triple) adj blind$).tw.

29. placebo$.tw.

30. prospective study/

31. or/12-30

32. case study/

33. case report.tw.

34. abstract report/ or letter/

35. or/32-34

36. 31 not 35

37. (exp animal/ or animal.hw. or nonhuman/) not (exp human/ or human cell/ or (human or humans).ti.)

38. 36 not 37

39. 11 and 38

Appendix 5

CINAHL search strategy

1. (MM "Endometrial Diseases+")

2. TX (endometr* N5 hyperplas*)

3. TX (endometr* N3 proliferat*)

4. 1 OR 2 OR 3

5. (MM "Metformin")

6. TX Metformin

7. TX glucophage

8. TX (dimethylbiguanidium or glucovance)

9. 5 OR 6 OR 7 OR 8

10. 4 AND 9

Appendix 6

PubMed search strategy

1. Endometrial Hyperplasia[mh]

2. (endometri* and hyperplas*)[tw]

3. (endometri* and proliferat*)[tw]

4. or/1-3

5. Metformin[mh]

6. metformin[tw]

7. glucophage[tw]

8. (dimethylbiguanidine or dimethylguanylguanidine)[tw]

9. (dimethylbiguanidium or glucovance)[tw]

10. or/5-9

11. 4 and 10

12. randomized controlled trial[ptyp]

13. controlled clinical trial[ptyp]

14. randomized[tw]

15. randomized[tw]

16. placebo[tw]

17. randomly[tw]

18. trial[tw]

19. (crossover or cross-over or cross over)[tw]

20. or/12-20

21. animals[mh] not humans[mh]

22. 20 not 21

23. 11 and 22

Appendix 7

Google Scholar search strategy
Keywords include: "endometrium", "endometrial", "hyperplasia, "proliferation", "metformin"

Appendix 8

ClinicalTrials.gov search strategy
(endometrial OR endometrium) AND (hyperplasia OR proliferation) AND (metformin OR glucophage OR dimethylbiguanidine OR dimethylguanylguanidine OR glucovance OR dimethylbiguanidium)

Appendix 9

World Health Organisation International Trials Registry Platform search strategy
(endometrial OR endometrium) AND (hyperplasia OR proliferation) AND (metformin OR glucophage OR dimethylbiguanidine OR dimethylguanylguanidine OR glucovance OR dimethylbiguanidium)

Appendix 10

OpenGrey search strategy
(endometrial OR endometrium) AND (hyperplasia OR proliferation) AND (metformin OR glucophage OR dimethylbiguanidine OR dimethylguanylguanidine OR glucovance OR dimethylbiguanidium)

Appendix 11

LILACS search strategy
(endometrial OR endometrium) AND (hyperplasia OR proliferation) AND (metformin OR glucophage OR dimethylbiguanidine OR dimethylguanylguanidine OR glucovance OR dimethylbiguanidium)
